# Correspondence from Paris

**Published:** 1850-01-01

**Authors:** 


### CORRESPONDENCE FROM PARIS.

Although the clouds that hang over the political horizon do not wear quite so threatening an aspect as they did, still there is sufficient agitation and excitement to divert men's minds from those high pursuits which ennoble the human race. Medical science naturally suffers from the unwillingness which exists to enter upon any subject unconnected with the passing events of the day, hence its literature has no patronage, and even the discoveries which are unfolded by the industry or the genius of individuals, pass almost unregarded, and there is little of that stimulus which urges the ambitious and the active on to laborious research.

The first week in November the schools, the hospitals, and the Faculty of Medicine, commenced their annual career. An unusually large concourse of students has this year filled the great amphitheatre of the schools, and the first address of the session was made by M. Denonville; it consisted principally of an eulogium upon the great surgeon Blandin, so lately taken away by the hand of death; he described him as he was, a plain, simple, modest, almost timid man; a clear speaker, an exact observer, an indefatigable labourer. The lecture was somewhat monotonously delivered, and a great deal too long, but it was elegant, clear, and comprehensive; the anecdotes which closed the address were pathetic and touching, they were sufficient to awaken the drooping attention, and they drew down repeated plaudits, which likewise accompanied the finale. M. Berald, the president of the faculty, then delivered a short welcome to the pupils; he spoke in the highest terms of their humane and noble behaviour during the prevalence of the cholera, alluded to the honours that many of them had gained from the President of the republic, as a proof how highly their services had been estimated, and spoke with an affectionate emotion of those noble youths whose devotion had been such that they had fallen victims, some to the disease itself, others to the fatigue and self-forgetfulness which they had voluntarily submitted to.

There has been but little attempted in the psychological department of medical science—scarcely any new work has appeared, but numerous facts of importance have presented themselves, and observations continue to be made on isolated points, which when brought together, will be found of much value, as increasing our stores, and enabling us in happier times to turn them to advantage. Amongst the cases that have offered themselves, is one which exhibits much singularity, and although there have been occasionally similar ones, which under the name of hysteric paralysis, have been recorded, there has been but little attention paid to them, nor have they been studied with the care which has been given to that which occurred at the Hospital Saint Marguerite, under the care of M. Valleix. A young married woman, nineteen years of age, was seized with fever, accompanied by delirium. She entered the Hotel Dieu, under Majendie; her malady ceased under the application of ice to the head. Upon her recovery her hair fell, her teeth became black, her face florid, her sight progressively indistinct, her hearing confused, and at length her reason was affected, and a state of occasional mental alienation occurred. At the beginning of last year she was seized with paroxysms of the most distressing character; after several hours of general indisposition there was a determination of blood to the head and face, with congestion of the external vessels of the face; she fell down apparently lifeless, the eyes turned upwards, the limbs immovable, the hands convulsively clenched, the forearm immovably bent upwards. During three-quarters of an hour, or an hour, this state lasted, she retained a perfect consciousness of her position, and of all that was passing around her; she understood everything that was said, but could make no reply; if she attempted to do so there was an unintelligible stuttering; as the force of the attack declined she burst into loud fits of laughter, or else cried. The fit over, she complained only of headache, and of a general feeling of lassitude, which completely disappeared at the end of a couple of days. She had about eight attacks; they did not come on at regular intervals, she had three in the month of July, the last was in the month of January in the present year. On the 21st of that month, after having experienced considerable indisposition, nausea, cephalgia, and many distressing symptoms, about nine in the evening suddenly her tongue seemed immovable, the upper and lower limbs felt as if they were swollen, and in the course of a few minutes she could move neither her arms nor her legs ; articulation was totally impossible. She was immediately placed in a warm bath, and friction was employed. Three days after she recovered the use of her upper limbs, and her speech was restored, but the lower limbs remained completely paralysed, and thus sensibility was lost. Upon admission into the hospital the power of moving the lower extremities was found completely lost; the muscles were flaccid, pins might be run into any part of the legs and thighs, up to the head, without producing the slightest sensation, the sensibility of the trunk was unchanged, there was no pain, the pupils were dilated and scarcely to be acted upon. Opium, baths, friction, ether, were employed, and in the course of eight days, considerable improvement in her general state took place, watchfulness during the night, cephalgia, loss of consciousness, and attacks of her former malady came on, but these were put a stop to, and at length a complete recovery took place. Among the phenomena which show themselves in hysteria, paraplegia of this kind occasionally occurs. M. Londouzy observed it in nine cases out of forty-six, and in the same number he saw fourteen cases of hemiplegia. That the application of ice to the skull during cerebral fever may produce a general loss of nervous power has been supposed, and there seems some reason to believe that the high susceptibility to nervous impressions may, in a young female, give rise to the train of symptoms which were visible in this instructive case, upon which M. Valleix has given some interesting remarks.

At the meeting of the Academy of Science on the 19th of November, M. Wanner read a paper upon the employment of ice as a therapeutic agent in the cure of medical and surgical maladies. This subject is one of considerable interest, especially in the consideration of its effect in diseases of the brain, there is little doubt that it has been indiscriminately employed in cases of delirium which have been considered to be the effect of cerebral inflammation ; in the delirium which attends upon scarlatina its employment is most injudicious, and it is more generally the cause of death than the means of cure; it ought indeed to be borne in mind, that in very few instances is delirium in scarlet fever to be attempted to be relieved by any local application, its best remedy being a free evaporation by the skin, hence the relief that is almost instantaneously afforded by the affusion of tepid water.

The most important labour that has been made public is the history of cerebro-spinal meningites by Michel Levy, the chief physician of the Val de Grace, as it occurred there during the last and the present year : of sixty cases, thirty-one exhibit instances of rapid death by the natural progress of the disease, seven by hydrocephalus, twelve complete cures, ten uncertain; of the twelve complete cures eight were bled, five once, three twice; ten had leeches applied, and as 280 leeches were employed, each had upon the average sixty-eight. Of those who died quickly, twenty-two were bled, leeches were applied in twenty-seven instances, 1620 being used, so that upon the average each had sixty leeches. The action of purgatives was found very precarious. The objections against the employment of calomel are not borne out by these cases, and opium seems to be more efficacious than any other remedy. It is, however, quite impossible to condense the long series of observations made by distinguished clinical professors; but we must acknowledge the justice of the conclusion which has been arrived at, that cerebro-spinal meningitis is one of the diseases which as yet so baffles the most experienced followers of medical science, that we are almost powerless in those rapid cases to which the French give the expressive term of "foudroyant" or thundering. More frightful than cholera or typhus, it is fortunately confined within narrower limits, and does not attack, as do the other diseases, whole masses of the population at the same moment.

A singular individual is just dead here, whose case has formed a study for the romance writer, the historian, and the psychologist. Any stranger in Paris could not fail to be struck with the eccentric appearance of a venerable old man, who was to be seen in all the places of fashionable resort dressed in peculiar garb; in the day-time, a large straw hat surrounded by a flaunting red ribbon, tied in a huge knot; his coat and vest such as appear in the old Venetian pictures, silk stockings, shoes with large buckles—he was thus to be seen at all the morning amusements; but in the evening his gala-dress was distinguished by the most glaring colours—red waistcoat, red pantaloons, and large red slippers, the collar of some unknown order and its star decorated his person, whilst his straw hat had received additional ornaments—there were artificial pearls, steel buttons, glass beads, and various glittering gewgaws. On addressing this remarkable personage, he would answer either in the choicest Italian or the most perfect French. He conversed on musical subjects like an artist, on the ballet like a professor, and if classical subjects were touched upon, he showed a familiarity with all the great authors; if poetic, he poured forth the richest streams of the choicest verses of those who are the master-spirits of different ages. It was impossible not to be struck with the wonderful powers of the man; he was evidently a man of the highest education. This person was the last descendant of one of the first families in Italy, having lost his brother, who was one of the most distinguished theologians of Calabria, but a short period before his own death. The Count di Carnevale had been a conspirator, in the year 1789, against the King of Naples; he was arrested and confined, together with the celebrated Cimarosa, and here first was developed the malady which deprived him of the use of a portion of his reason. The account given by himself was, that the dungeon in which he was placed was exceedingly low, and that on one occasion upon which some news was brought to him, he suddenly rose from a sitting posture and struck his head with considerable force; a concussion of the brain followed, which was relieved after a long illness by judicious medical treatment. He was banished, and sought Paris as the place of his residence. His property being confiscated, he was compelled to give lessons for his support, and was progressing much to his satisfaction in his undertaking, when he was plunged in the deepest distress by reading in one of the French newspapers the death of a lady to whom he had been early in life attached, and to whom he had been betrothed, but who had been compelled by the king to marry another person. From this moment disease seems to have preyed upon his mind; but whilst his memory and his judgment upon all the ordinary circumstances of life were unimpaired, a singular hallucination disturbed all his peace, and a fixed idea took possession of him, which no subsequent events of his life could eradicate. He believed that, whilst seated in his room, the lady of his affections suddenly appeared before him, told him that she was not dead, that it was her husband, and that a mistake in the announcement of the newspaper had already caused her much misery, as it was universally believed. She came, she declared, to offer her hand and her heart. He accepted these proofs of her truth, and made every preparation for his marriage. The most costly fête was prepared for the occasion, no expense was spared; the persons of whom he ordered the necessary requirements furnished them without hesitation on learning the name of the bride, but nobody had yet seen her; every one felicitated him on his coming happiness, and all waited with anxiety to pay their respects to this paragon of females—yet did she not appear amongst them. At length, after waiting for some time, suspicions being aroused, his friends were determined that an explanation should be arrived at, that they might no longer be kept in suspense. It was soon evident that it was a delusion—that it was only an imaginary being that had appeared, and the truth was confirmed on inquiring at Naples after the lady. She was dead, and all that had been reported was true. It was impossible to convince the unfortunate nobleman that he was labouring under a delusion; if an attempt was made to undeceive him, it brought on paroxysms of rage. It was at last found absolutely necessary to consign him to a lunatic asylum. At Charenton, by the humane attention of the physicians, he was partially restored to his reason, but hallucinations upon the subject of his approaching marriage still remained; he was always dressed in the costume that he believed best adapted for the marriage ceremony; he was for ever waiting his coming bride; perfectly harmless, he was always welcome wherever he went. The first two or three days that he exhibited himself in his picturesque dress, he attracted crowds; curiosity gradually subsided, his story became known, and every one exhibited sympathy for him. He slept completely dressed, and generally rose about four in the morning to prepare himself for the bridal fête. Whenever he felt a paroxysm come on, which had been the case since he had been excited, he went to the hospital, and remained there until he recovered. He could not, towards the end of his career, believe that death ever really occurred. It was one of the sweetest delusions that could render the life of such an unfortunate being supportable. He never could reconcile it to his mind that any person of merit ever could die; he thought that they continued to live upon the earth, though invisible to the greater number of mankind. He would sometimes speak of his having met Mozart or Madame Malibran; and with tears in his eyes, and his hand on his heart, would pathetically remonstrate with any one who seemed to doubt the fact. He continued to receive a great deal of money as an Italian master; he divided it into three portions, one for his own necessities, another for the poor, and another for his wedding garments. Upon these he would ruin himself, if not gently admonished by one of his friends, for he would purchase the richest velvet and the most costly ribands. Many anecdotes of his delicacy and his integrity are related. He was invited to the houses of some of the most distinguished men of the day, and nobody could believe, excepting from the extravagance of his costume, that he was the frequent inmate of a lunatic asylum. His death was instantaneous; he was taking his accustomed stroll on the Boulevards, when he fell, and with a gentle sigh he gave up his harmless but hapless life. He was quickly surrounded by the passers-by, who, recognising at once their old friend, bore him along to the Hôpital Beaujon. The first inquirer after him was the amiable wife of the accomplished Lablache, who had heard of his sudden seizure. The intelligence was quickly spread to the Opera-house, where he had always been welcomed with a free admission, and all the artists determined on paying the last sad tribute of respect to their old friend by accompanying him to his grave. Fiorentino wrote a little memoir of him, and we are promised a still longer narrative of his singular life.

A curious investigation has been going forward as to the paralysis produced by the application of cerussa, or white lead. It appears that by the precautions taken in the manufactories, the work-people in the neighbourhood of Rouen escape from its bad effects, but that those in the neighbourhood of Paris are more affected by them. We are about to have a statistical report from the different hospitals, at the wish of the Académie de Médecine. It is said that the paralysis seldom or never is accompanied by any alteration in the mental faculties, except occasionally in some instances, where belief of suddenly-acquired wealth has occurred.

M. Bouchut is preparing for publication his observations on the softening of the brain, observable in persons of advanced years. He read, at a meeting of the medical society of the hospital, some notes which he purposes to expatiate upon. He has, in all his pathological researches into the nature of the malady, seen incrustations formed upon the sides of the arteries of the brain, and fibrinous coagula. These lesions have naturally had the effect of diminishing the diameter of the vessels, and consequently impeding, especially in the smaller vessels, the circulation: he has counted no less than fifty-four of these coagula, so that the canal has been reduced to two-thirds its ordinary size; they are situated between the internal and the middle tunics; they are found in the basilary trunk, and they there destroy the internal membrane. The incrustations are white, cartilaginous, and differ from other incrustations, which are calcareous, and composed of molecular granules. The coagula are small, floating, and not adhering to the walls of the vessels. From such facts he arrives at the conclusion, that a want of due nutrition of the brain is the cause of its becoming soft, and that it also tends to produce the senile gangrene of the brain, to which attention has been drawn by several pathologists, and more especially by Sir Astley Cooper and by Magendie.

M. Bondi has described an interesting case of mania, periodically returning, which is exceedingly well worth recording. It occurred in a youth now of twenty-one years of age, of a sanguineous habit. He was suddenly, and without the slightest previous indication, seized, in the sixteenth year of his age, with delirium, unaccompanied by fever. He destroyed everything around him. From the 19th of July, 1840, to the 21st of August, he continued in an alarming state; the only consequence was a debility of body, and also of his intellectual faculties; and his health became sufficiently re-established to enable him to follow his usual employment in the fields. On the 19th of July of the following year he had precisely a similar attack, and for four successive years he had the same kind of paroxysms; for these he was usually bled, and purgatives administered, which completely relieved him for the time. In the year 1844, M. Bondi saw him: he was then in one of his paroxysms, his countenance flushed, and apparently angry. He had hallucinations of sight; his pulse was small; his tongue covered with fur; his skin burning. There was a remarkable tension over the region of the liver. He was exceedingly loquacious, although under ordinary circumstances he was taciturn. His appearance was very much that of a Cretin. He was of short stature, had a low forehead, the summit of the head was depressed, his nose flat, and his mouth large. He had a sister of twenty-five years of age, epileptic and idiotic. M. Bondi bled him, gave him forty grains of scammony, applied leeches behind the ear, and to the anus. This had some effect upon him; but his paroxysms lasted some days, during which he attempted to throw himself into a tub of water. This seeming to M. Bondi to be an indication of nature, he ordered cold bathing, and cold affusion upon the head, prescribing hellebore. After this treatment a change took place; the patient became perfectly tranquil, but was seized by a religious monomania, which seemed to soothe and to calm him. Sulphate of quinine and acetate of morphia were administered. After a few doses had been taken, all the symptoms of an intense fever supervened. The cerebro-spinal system seemed to be in a state of collapse. A large blister was then applied to the nape of the neck, and advantage was taken of the denudation of the skin to try the effect of endermic medication: the sulphate of quinine was thus externally applied and taken into the system. The effect of this treatment was prompt and decisive; the young man was speedily restored, and was enabled to return to his labour; and from that period up to the present moment—now four years—he has had no return of his former attacks. The precaution has been taken to bleed him, and to administer a drastic purgative, just previous to the usual time for the coming on of the paroxysm. It is to be hoped that, the periodicity of the disease being interrupted, there is now no probability of its return.

M. Verga has published in one of the Italian journals a case in proof of the extreme insensibility of the insane to suffering, even when an internal alteration is taking place in the structure, and which, under ordinary circumstances, must give rise to excruciating suffering. An insane person ate and drank heartily to the last day of his life; he died under a violent paroxysm of asthma. Upon examination after death, it was found that there was a most extensive ulceration of the stomach. The disorganization had advanced to a striking extent, without its having apparently had the slightest effect upon the individual.

M. Bizzi has given an instance of the softening of the anterior lobe of the brain, the result in the patient, whilst living, being the loss of speech. Upon this case, and upon similar ones, M. Strambio has made some very ingenious comments.

M. Verga has also furnished us with some interesting views of pellagra, and of the paralysis to which the insane are subject. He carries out the doctrines of Baillarger, and agrees with him upon the most material points.

On the 25th of November, a most interesting ceremony took place at the Asylum of the Deaf and Dumb. It was the anniversary of the birth of the virtuous and good Abbé de l'Epée, to whom society is so much indebted for having first directed the public attention to those unfortunate beings who, deprived of the sense of hearing, had hitherto been entirely neglected, and left to the miseries which their privation exposed them to: for, notwithstanding we are willing to give to Pereira, whose claims have been so admirably set forth by Seguin, all the praise that is due to him, for having been the first who recognised the power of educating the deaf and dumb, and of distinguishing them from idiots, and individuals of retarded mental power, we must look to the Abbé de l'Epée as the first person who brought the subject before the world, and as one who indefatigably pursued his object until he obtained means, from the highest quarters, of establishing institutions, which do honour to the human race. In remembrance of him, the deaf and dumb from all quarters of France assembled at a dinner. They were surrounded by their instructors, by their medical friends, and by several persons who take an interest in their welfare. A deaf and dumb gentleman, of the name of Berthier, who has lately obtained the distinction of being made a member of the Legion of Honour by the President of the Republic, in consequence of his literary talent, presided over his co-mates in deprivation. He proposed several toasts, rendering himself completely intelligible by his signs and his pronunciation of sounds, which were recognisable by the attentive eye of those who were acquainted with the means by which information is conveyed from one to the other. Several healths were drank, some of them containing slight political allusions to the interest they felt in the preservation of order; others were in compliment to those who were present, or who had been enabled to render some service to the good cause. Notwithstanding the meeting was such, that even a follower of William Penn, or the more modern leaders of the sect of the Quakers, might have envied the silent tranquillity of the scene, there was considerable hilarity and enjoyment: if there was little interchange of soul by the medium of the tongue, there was the beaming eye of gratitude, and the expressive change of feature which bespoke contentment, and the development of the better feelings of the heart. The company broke up at an early hour, promising to each other a repetition of the pleasure upon another anniversary of the birth of the excellent man, to whom each was indebted for having rendered life endurable. The institutions for the deaf and dumb throughout France are upon an admirable system; there is an annual publication given out under their authority, which narrates every improvement that has occurred within the year, collecting from every source, domestic and foreign, hints as to the treatment followed, and making valuable suggestions for the benefit of the sufferers in general.

At a meeting of the Academy of Sciences, Arago announced that at length a remedial agent had been discovered, which would completely cure hydrophobia. He stated that M. Roger de Hericourt had discovered, during his travels, a tree, the bark of which was an infallible remedy, and that dogs that had exhibited the most marked symptoms after being bit, had completely recovered under its use. It would seem, however, that where the human system has become impregnated with the virus, and the disease has been fully developed, there is not the same certainty of recovery. A committee has been appointed to confer with M. Roger de Hericourt, and it is expected that, at the next meeting, there will be a full explanation of the discovery to which he lays claim.

Suicides have been very frequent, within the last few months, in Paris; and besides political events, there have been such extraordinary changes in domestic life, that such events cannot be considered surprising, where there exists any predisposition to insanity, upon which most psychologists seem now to concur in believing that a tendency to the commission of suicide depends. It is singular, but it has been observed, that whilst plunging into the river is the more usual means had recourse to in the summer, that during the winter season, suffocation from the fumes of charcoal is the more ordinary thought that crosses the suicide's mind. Instances of throwing themselves from high places have been of late very common amongst the unfortunate persons who have been impelled to the fearful termination of life by self-destruction. The column in the Place Vendôme, like the Monument in London, has been calmly ascended by persons who, on arriving at the summit, have escaped the vigilance of the guardians, and have cast themselves down. During the last few months, there have been an unusual number of these instances; and as there is no coroner's inquest, nor any public inquiry, we are left in ignorance of the motives, and become only incidentally acquainted with the facts, and they are seldom commented upon by the public press, even when mention is made, which is not always the case, of such a fearful occurrence.

The favourite actress, whose hypochondriasis was the subject of a communication in one of the former numbers of the *Psychological Journal*, has, after the judicious treatment of her physician, and a prolonged retirement in the country, been enabled to resume her theatrical career, to the great delight of the Parisian audiences. Her life and animation draw down thunders of applause, and her gaiety is the source of hilarity and enjoyment; yet does she still occasionally suffer from that state of depression to which actors have been liable, and from that species of hypochondriasis which, according to Leuret, attaches itself to those who appear the happiest. Intellectual labour of any kind, too long persevered in, produces this malady in a gentle form; but where an apparent gaiety is to be assumed, the reaction is of a terrific character.

The cholera has at length altogether ceased its frightful ravages, both in Paris and in the departments. Every remedy that human ingenuity could suggest has been tried, with as little success here as in other parts of Europe. The attention that was given to it by the higher authorities, led to the greatest care in all the public establishments, and to the maintenance of cleanliness, and the purification of the more unwholesome part of the city. Amongst the poorer classes the mortality was greater than amongst the wealthier; still this did not bear the same proportion as in London. The military escaped better than the civilians, as much from the care that was taken to support their moral courage, as from the zeal and assiduity which the officers exhibited in frequently removing their men, from time to time, from their positions, and changing frequently their quarters. There was a Board of Health established, which took the precautions, as far as was possible, of instructing the people as to what steps they should take, on being first warned by the premonitory symptoms of the approach of the disease. Amongst the works that were translated from different languages for the occasion, was the pamphlet written by the Editor of the *Psychological Journal*, under the title of "*The Cholera considered Psychologically.*" This translation was undertaken by M. Colmache, a young surgeon, who promises to take a high position in his profession, the son of the much respected and learned secretary of Prince Talleyrand. He has only to walk in the steps of his father to gain the good opinion of all. The pamphlet is remarkably well translated; it conveys the opinions and the recommendations of the original author in clear and concise language, and has obtained considerable approbation from those who are the best judges of the intrinsic merits of a book. Such translations are of much use; they exhibit the train of thought which pervades another country, and give a knowledge which otherwise would be circumscribed within very narrow limits. Numerous have been the pamphlets laid upon the table of the Academy of Sciences, which have been written in consequence of the cholera, for it has awakened the old question of contagion.

Various trials have been made of the power of electro-magnetism upon the nervous system, the results of which have been, from time to time, made known by M. Sequard. The contractility of the tissue, of which the dermoid coverings are composed, depends upon the nervous system, and it is a consequence, that should they be acted upon the muscular tissue would also be affected, notwithstanding the opinion of Müller and of Henle, that there is an essential difference in their organization. The skin of the scrotum was selected in the first instance, as its connexion with the dartoid muscle would be to render the action more convincing. The contraction was found rapid. Deep and numerous folds were produced; and vermicular and undulatory movements followed; but what showed that the whole nervous system came under the control of the electro-galvanic apparatus, was the production of a pleasurable sensation. When tried upon other portions of the body, especially upon the back of the arm, the goose-skin roughness was produced. Some individuals are scarcely susceptible of the action, whilst in others it produces great impression; but what appears most singular is, that in certain paralytic cases the electro-galvanic apparatus acts with the greatest intensity, giving rise to the production of small elevations over the whole surface of the skin. It has likewise been tried for the purpose of awakening the energy of idiotic and of helpless persons, and for a short period it produces an apparent effect; but as it seems to leave little influence behind, whilst it produces momentary terror, its use has not been persevered in. M. Brown Sequard is following up a train of experiments upon the influence of electro-magnetism, which are placing him in the highest rank of physiologists. He has also given some curious observations on the reproduction of the sciatic nerve, and has confirmed the views of Fontana, of Tiedemann, and Flourens. The *Journal of Psychology*, which was to have re-appeared in October, has not been published. It is to be regretted that no patronage can be obtained for this interesting work. There is, however, now some little prospect of the recovery of science, for Dumas, whose name is so well known in England as a scientific chemist, has been appointed minister of public works, and already has he shown much zeal in contributing towards the progress of arts and of science. He has appointed a commission for the prosecution of useful public works; he has given orders for the establishment of baths for the poor; and he is about to forward the views of those who are urging an increase of establishments for invalids and for the aged. It is to be hoped that there is a dawn of prosperity after the long darkness in which everything has been plunged; and that more especially the sciences connected with the healing art will meet with that encouragement which they so richly deserve.

Some attempts are making to obtain advantages for the publishers and booksellers, who have been suffering from the total stagnation which has so long existed in literature. They have been assisted in some small degree by a public lottery, which embraces the works that have been recently published; but it is not upon such a scale as to be of any very great service. Medical literature has suffered materially, whilst political works have flourished beyond all former example. Lamartine's monthly periodical finds more than one hundred thousand subscribers, whilst the *Annals of Medicine* cannot obtain two hundred. Quackery, however, in all its various branches, succeeds, in spite of some excellent articles in the *Gazette Médicale,* which quotes the clever saying of Guy Patin, "If all the quacks were hung, cord would become a very dear article."

---

